# Postural assessment of patients with non-conventional knee endoprosthesis

**DOI:** 10.1590/1413-78522014220500826

**Published:** 2014

**Authors:** Luciana Nakaya, Liliana Yu Tsai, Reynaldo Jesus-Garcia, Marcelo de Toledo Petrilli, Dan Carai Maia Viola, Antonio Sérgio Petrilli

**Affiliations:** 1 Universidade Federal de São Paulo, Instituto de Oncologia Pediátrica, São Paulo, SP, Brazil, Instituto de Oncologia Pediátrica, Grupo de Apoio ao Adolescente e à Criança com Câncer da Universidade Federal de São Paulo- IOP-GRAACC, UNIFESP, São Paulo, SP, Brazil

**Keywords:** Osteosarcoma, Posture, Evaluation, Physical therapy specialty

## Abstract

**Objective:**

To investigate the correlation between the sagittal and frontal alignment and possible postural asymmetries found in patients submitted to total knee stent placement for osteosarcoma.

**Methods:**

Twenty two individuals were divided into two groups according to tumor location: femur group (13 patients) and tibia group (nine patients), who were evaluated through postural analysis software (SAPO).

**Results:**

No statistically significant difference was found between groups, supporting previous result showing that both groups present the same postural asymmetries.

**Conclusion:**

We conclude that both groups have the same postural imbalances, especially the knee of the affected limb that presents hyperextension and center of gravity shifted anteriorly and laterally to the non-affected limb, indicating changes in weight bearing and influencing the gait pattern and balance. *Level of Evidence II, Prospective Comparative Study.*

## INTRODUCTION

Childhood cancer in Brazil comprises the third leading cause of morbidity and mortality among the population of 1-14 years old.¹ Primary bone tumors occupy the sixth place in frequency among all malignant tumors of childhood, since young adults are presented in third place.² Among these tumors, osteosarcoma stands out representing approximately 60% of cases, and there are an estimated 350 diagnoses of osteosarcoma per year in Brazil.^3^^,^ 4

Osteosarcoma (OS) occurs in the second decade of life being more prevalent in boys, affecting mainly bone metaphysis, being distal femur the most common primary site followed by the proximal tibia region. In recent years there has been great improvement in the prognosis and quality of life of these patients due to significant advances in orthopedic prosthesis and conservative surgeries. Prior to the 70s, these patients were treated solely with surgery, systemic recurrence occurring in over 50% of cases in less than six months and 90% of deaths due to progression of the disease.² From several randomized studies the importance of the association of adjuvant therapies such as chemotherapy was demonstrated, which significantly improved cure rates. The effect of adjuvant chemotherapy is important for limb preservation procedures, since chemotherapy alone improves the prognosis by reducing the size of the tumor and, thus, surgical margins.^5^

When the resection of an osteosarcoma involves a structural or functionally important bone it is necessary to have a bony reconstruction in order to restore function and stability of the resected bone. Two types of surgery are performed to control OS: amputation or limb preservation surgery, the later consists of tumor resection with bone reconstruction from an endoprosthesis, homograft segment from a bone bank, by bone transport or a combination of methods.^5^^,^ 6 It should be remembered that the limb preservation surgery is only indicated when it ensures the patient a survival rate exactly to or better than amputation.^6^ Surgery for placement of total knee endoprosthesis in patients with tumor located at the end distal femur consists of resection of the tumor, with maintenance of the extensor mechanism of the leg, while for patients with tumor located in the proximal end of the tibia, it consists of tumor resection followed by detachment of the patellar tendon and reinsertion in the prosthesis itself or in a folded flap of the gastrocnemius muscle.^7^ in a study by Tsai *et al*,^7^ it was found that patients with distal femur OS show better results in the following categories: amplitude of motion, muscle strength and gait. The best functional results are strongly correlated with maximum preservation of structures,^5^ therefore, the limb preservation procedures have become important for the treatment of osteosarcoma by presenting more functional and psychological benefits compared to amputations.^8^

There is little information on the damage and physical disability of these patients after surgery with endoprosthesis placement, however, when cancer treatment ends, these patients, unfortunately, have a physical disability due to implantation of the prosthesis, which can be prejudicial in his personal and social life. Therefore, the assessment of postoperative functional outcome is becoming very important to the quality of life of this population that has increased survival rates. However, these patients who underwent prosthesis placement have more complications, such as infections, wear of some of the components, fractures or dislocations, requiring revisions surgeries.^9^^,^ 10

Because of all the changes resulting from the surgical procedure, these patients also undergo postural and biomechanical adaptations. As this type of tumor affects mostly teenagers, and they are still in the growth phase, it is important to pay attention to possible bodily asymmetries that can directly influence their functional level. A postural assessment is a widely used method to understand the alignment of body segments, from which we can obtain information about the functionality of the individual to enable treatment plans that would stimulate their abilities. There are several qualitative and quantitative methodologies that allow postural assessment of both children and adults. In Brazil, software has been specifically developed for postural analysis, named SAPO (*Software for Postural Assessment*). It has established measurement protocols, but also allows users to organize their own protocol and conduct free measurements. SAPO generates an Excel report on posture from information derived from the coordinates of the patient's anatomical points.^11^ This postural assessment software was validated, presenting itself as a reliable tool to measure accurately body distances and angles.^12^

Postural analysis is one of the first physiotherapy assessment tools, since the presence of postural asymmetries may contribute to improper weight distribution and muscle recruitment, leading to clinical implications. Therefore, postural assessment becomes important to build a treatment strategy, leading the individual to its desired goal.

This study was approved by the Ethics Research Committee of Universidade Federal de São Paulo. The authors declare no conflict of interest. The aim of this study was to verify the presence of correlation between the alignment in the sagittal and frontal plane and possible postural asymmetries found in patients who underwent surgery for placement of total knee endoprosthesis due to osteosarcoma (total ENCJ).

## MATERIALS AND METHODS

This is a cross-sectional study, and the selection of participants was performed by convenience.

The group was formed by 22 individuals who underwent surgery for total ENCJ placement, regardless of gender and age above 12 years old. The groups were divided into: femur group (FG) and the tibia group (TG), according to tumor location. (Table 1)

**Table 1:** Characterization of test subjects


	Number of subjects	Feminine	Masculine	Mean of current age (years)	Mean PO (years)	Lower limb affected
Femur Group	13	5	8	21.76 (±5.24)	4.53 (±4.23)	RLL (7)LLL (6)
Tibia Group	9	2	7	26.77 (±5.26)	8. 88 (±4.59)	RLL (6)LLL (3)

RLL: Right lower limb; LLL: Left lower limb.

This study was conducted Physiotherapy Session, Instituto de Oncologia Pediátrica (IOP), Universidade Federal de São Paulo (UNIFESP), São Paulo, SP, Brazil.

Inclusion criteria: patients with at least one year postoperatively of both genders with osteosarcoma in the distal femur or proximal tibia who underwent tumor resection and placement of total knee endoprosthesis.

Exclusion criteria: patients who showed no static equilibrium in the standing position; patients with relapsed or progressive disease.

The study offered no risk or harm to participants. Its methodology included non-invasive and painless procedures. The participant or his responsible guardian could interrupt the procedure if necessary. The participant or his guardian may request at any time the termination of the research, without any financial or material damage. The study was prepared in accordance with the Guidelines and Rules of Research Involving Human Subjects (Resolution 196/1996 of the National Health Council). The project was approved by the Ethics Research Committee of Universidade Federal de São Paulo, São Paulo, SP, Brazil (CEP 1747\10).

As a benefit, the study contributed to the postural assessment of this population, allowing a greater understanding of postural changes in the long term.

For data collection the following material was used:

To characterize the participant and authorizing his/her participation: anamnesis and free informed consent form.

For postural assessment: a digital camera, a tripod, a plumb line marked with two Styrofoam markers delimiting one meter, markers made with Styrofoam balls two inches in diameter for fixing the anatomical landmarks of the participants, tape, ruler and the Software for Postural Analysis - SAPO.

Participants to this study were selected according to inclusion and exclusion criteria established by the researcher. Subject and/or to legal guardians were invited and after agreement to participate, they completed and signed the consent form, which describes all the information about this study. If the subject and/or guardians could not read, the reading of the term was made by the researcher.

Then, evaluation of the participant was performed, which comprised two steps: anamneses and postural assessment.

Through the anamnesis form (Annex 1) personal data and characterization of participants was collected, such as history of previous illness, frequency of physical therapy, surgery date, and stage of cancer treatment.

**Chart 1:**
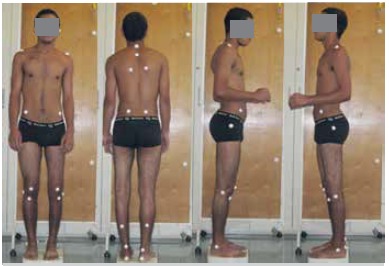
Location of anatomical points for postural analysis.

Postural assessment was done by marking anatomical landmarks of the body of each individual using as markers little styrofoam balls cut in half and previously prepared with double-sided tape, which were fixed at specific anatomical points. (Chart 1) A plumb line marked with two Styrofoam balls located one meter from one another, to enable calibration of the photo in SAPO software. We used a digital photo camera positioned on a tripod at 90cm and at a three meters distance from the participant.

**Figure 1:**
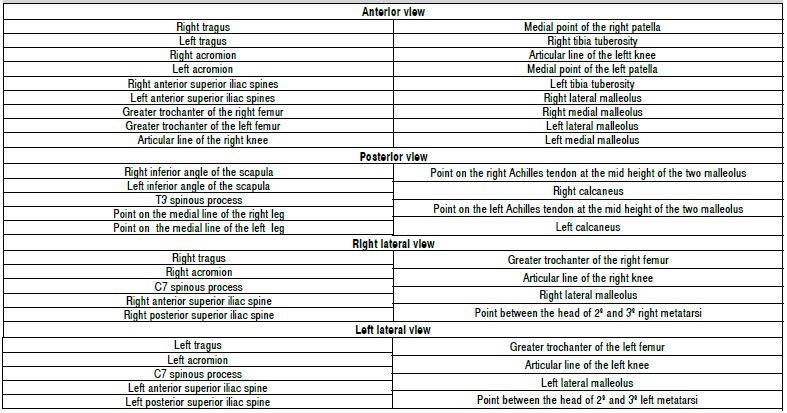
Procedure for postural evaluation resulting in photographs of participants in anterior, posterior and right, and left lateral views.

After labeling the anatomical points the participant was positioned standing next to the plumb line and he/she was requested to change position in order to allow the four views (anterior, posterior, lateral right and left) to be photographed. (Figure 1) After registration the photos were downloaded to a computer and analyzed by SAPO software.

Postural assessment allowed the measurement of the variables listed in Table 2, measured for the femur and tibia groups.

**Table 2:** Variables of postural analysis.

Head horizontal alignment	HHA
Acromions horizontal Alignment	AHA
Horizontal alignment of anterior superior iliac spines	HAAIS
Angle between the two acromions and the two anteriorsuperior iliac spines	A_HAAIS
Difference in limb length	DLL
Alignment in the frontal plane	AFP
Alignment in the sagittal plane	ASP
Q angle	Q _Â
Vertical alignment of the head	VAH
Knee Angle	K_Â
Ankle angle	A_Â
Horizontal alignment of the pelvis	HAP

### Data analysis

We used the *Student* *t* test for independent samples on the results of the postural evaluation for comparison between femur and tibia groups for the following variables: HHA, AHA, HAAIS, A_HAAIS, DLL, AFP, ASP, Q _Â, VAH, K_Â, A_Â, and HAP. Additional analysis also using the Student t test for all the variables of the postural evaluation was performed as well, however, using only the data from participants with compromised right lower limb both the femur and tibia groups, in order to check for possible correlation. All analyzes were performed using SPSS 12 software, considering a significance level of 5%

## RESULTS

We present the results obtained using the methodology previously described, in order to verifying differences between the femur and tibia groups regarding postural evaluation.

Table 3 illustrates the mean, standard deviation and t-test of the angles of the various variables obtained through the SAPO protocol.

**Table 3:** Mean, standard deviation and t test of the variables obtained by postural assessment between the femur and tibia groups.

**Variables**	**Side**	**Femur group**	**Tibia group**	**t teste (value of p)**
HHA		0.26º (1.26)	0.42º (1.35)	0.720
AHA		0.65º (0.70)	1.16º (0.85)	0.647
HAAIS		1.87º (1.05)	0.92º (1.05)	0.546
A_HAAIS		1.21º (1.33)	- 0.21º (1.32)	0.470
DLL		-0.7 (0.59)	-0.87º (0.60)	0.856
AFP		1.25 (4.19)	0.78 (7.92)	0.955
ASP		37.51 (4.99)	43.47 (3.37)	0.390
Q _Â	Right	29.98º (4.04)	20.53º (4.04)	0.126
	Left	28.38º (3.33)	24.35º (4.10)	0.452
VAH	Right	18.15º (4.34)	20.82º (4.81)	0.688
	Left	22.24º (2.32)	17.86º (4.42)	0.352
K_Â	Right	-0.9º (2.30)	-3.43º (2.66)	0.483
	Left	0.33º (1.84)	-4.6º (1.73)	0.077*
A_Â	Right	85.79º (1.43)	86.06º (1.62)	0.905
	Left	85.75º (1.45)	87º (1.07)	0.531
HAP	Right	-9.98º (1.49)	-10.23º (2.67)	0.929
	Left	-9.93º (0.94)	-11.36º (3.00)	0.606

* (p < 0,05).

The t test indicated no significant difference between the femur and tibia groups for all variables of postural analysis, indicating that the same postural changes are present in both groups. There was a strong tendency for the variable left K_Â, with p = 0.077.

From the characterization of participants, we noticed a predominance of the right lower limb (RLL) to be more committed in both groups. Therefore, we also performed a comparison between the femur and tibia groups, using instead, only the data of patients who had affected RLL. Thus, in the femur group D = 7 patients, and in the tibia group D = 6 patients. Table 4 shows the average and standard deviation and t test of the variables of postural evaluation only for patients with affected RLL in both groups.

**Table 4:** Mean, standard deviation and t test of the variables obtained by postural evaluation between the femur and tibia groups considering only affected RLL.

**Variable**	**Side**	**Femur Group R**	**Tibia Group R**	**t test**
HHA		0.09º (1.19)	-0.98º (1.81)	0.621
AHA		0.37º (0.60)	1.56º (0.67)	0.270
HAAIS		3.47º (1.60)	2.6º (0.80)	0.654
A_HAAIS		3.07º (2.05)	1.07º (1.01)	0.424
DLL		-1.61 (0.94)	1.77 (0.57)	0.897
AFP		-8.74(4.05)	-11.55 (5.77)	0.692
ASP		32.96 (6.70)	44.23 (5.01)	0.217
Q _Â	Right	26.36º (5.35)	17.35º (4.36)	0.228
	Left	30.61º (5.93)	27.38º (5.49)	0.701
VAH	Right	23.10º (5.44)	18.25º (6.82)	0.585
	Left	24.51º (12.25)	14.6º (15.81)	0.119
K_Â	Right	0.2º (2.86)	-1.22º (3.42)	0.755
	Left	0.36º (3.04)	-5.63º (2.41)	0.160
A_Â	Right	85.4º (1.85)	85.63º (2.37)	0.939
	Left	86.91º (1.71)	86.28º (1.47)	0.788
HAP	Right	-12.53º (2.08)	-12.48º (3.51)	0.991
	Left	-10.39º (1.25)	-11.08º (4.03)	0.863

* (p < 0,05).

No statistically significant differences were found when comparing the groups using only participants with affected RLL, reinforcing the result that both groups have the same postural asymmetries.

## DISCUSSION

There have been included in this study patients who underwent surgery for limb preservation with unconventional placement of total knee endoprosthesis, and they were divided into two groups according to tumor location (femur and tibia groups). Both groups showed postural asymmetries, but there was no statistically significant difference between variables of postural analysis. These asymmetries are found due to the fact that osteosarcoma affects a population in the second decade of life², i.e. in adolescence, and therefore, these individuals are in the process of bone and other structures growth. The surgical placement of total knee endoprosthesis is one of the techniques used for local control of osteosarcoma and it includes withdrawal of epiphyseal growth of both the femur bone and tibia bone. Thus, one can understand the fact that this population is prone to postural changes.

The discrepancy between lower limbs (LLs) is the most frequent finding in the literature. According to Yoshida *et al.*,^13^ children who undergo limb preservation surgery for malignant bone tumor of the lower limbs face several postoperative problems during growth, in particular differences in the limbs length and losening that may cause serious functional disorders. In this study, both groups showed a difference in length of the lower limbs, being the affected limb always the shorter. Thus, the whole hemibody of the affected limb will show changes that occur due to this asymmetry: depression of the scapular waist and inclination with anteversion of the pelvic girdle.

One of the variables of postural analysis, the left knee angle presented a biased p value (p=0.07). It represents the bending angle (positive values) or extension angle (negative values) of the knee. In this study we found similarity of both groups presenting knee hyperextension of the affected lower limb. This finding corroborates the study by De Visser et al.^14^ reporting that the operated lower limb has lost muscles and knee ligaments leading to loss of proprioceptive input, a fact which contributes to knee hyperextension, which also occurs as a sort of protection mechanism and safety during gait, especially in the stance phase. It should be taken into account that patients were analyzed barefoot, and most of them use compensation in footwear due to lower limb discrepancy.

Finally, another finding of this study concerns the center of gravity (CG) presented by this population. In both groups CG is displaced anteriorly and lateralized to the contralateral to the operated lower limb, suggesting an inadequate weight discharge of the lower limbs, that may interfere with the gait and balance pattern. This finding agrees with the results found by Carty *et al.*,^15^ who concluded, through an electromyographic study that this population presents gait alterations due to inadequate muscle activations especially the rectus femoris and hamstrings, and they, therefore, suggest that rehabilitation should focus on reprogramming the gait pattern. In his other study,^16^ the author concluded that the gait of patients with knee endoprosthesis shows a pattern that reduces the "momento" of the knee and hip in the affected lower limb, suggesting compensation for pain, stability and\or muscle weakness. Visser *et al.* 17 in their study on the balance of this population concludes that the balance in the standing position is good, but is negatively affected the more visual and cognitive difficulties are being imposed, showing that the level of postural automatism of these patients is not yet complete.

This study was limited by the small sample size in the groups, and although the results were biased, there is a need for a more extensive study to increased statistical strength of the variables.

## CONCLUSION

We conclude that, by analyzing postural evaluation by photometry, the femur and tibia groups exhibit similar asymmetries in the operated limb. Regarding the knee angle, both groups perform knee hyperextension on the affected lower limb, a fact that is directly related to the loss of proprioceptive input in the region. Furthermore, these patients have altered CG demonstrating inadequacy of weight bearing on the lower limbs, which directly affects the gait and balance patterns. Therefore, it is suggested that the rehabilitation of these patients should focus on better weight distribution and proprioceptive training of the lower limbs, so that therapeutic goals are achieved with greater success.
